# Rényi Entropy-Based Spectrum Sensing in Mobile Cognitive Radio Networks Using Software Defined Radio

**DOI:** 10.3390/e22060626

**Published:** 2020-06-06

**Authors:** Ernesto Cadena Muñoz, Luis Fernando Pedraza Martínez, Cesar Augusto Hernandez

**Affiliations:** 1 Systems and Industrial Department, Universidad Nacional de Colombia, Bogotá 111321, Colombia; 2 Telecommunications Engineering Department, Universidad Distrital Francisco José de Caldas, Bogotá 110231, Colombia; lfpedrazam@udistrital.edu.co; 3 Electrical Engineering Department, Universidad Distrital Francisco José de Caldas, Bogotá 110231, Colombia; cahernandezs@udistrital.edu.co

**Keywords:** mobile cognitive radio networks, Rényi entropy, software defined radio, spectrum sensing

## Abstract

A very important task in Mobile Cognitive Radio Networks (MCRN) is to ensure that the system releases a given frequency when a Primary User (PU) is present, by maintaining the principle to not interfere with its activity within a cognitive radio system. Afterwards, a cognitive protocol must be set in order to change to another frequency channel that is available or shut down the service if there are no free channels to be found. The system must sense the frequency spectrum constantly through the energy detection method which is the most commonly used. However, this analysis takes place in the time domain and signals cannot be easily identified due to changes in modulation, power and distance from mobile users. The proposed system works with Gaussian Minimum Shift Keying (GMSK) and Orthogonal Frequency Division Multiplexing (OFDM) for systems from Global System for Mobile Communication (GSM) to 5G systems, the signals are analyzed in the frequency domain and the Rényi-Entropy method is used as a tool to distinguish the noise and the PU signal without prior knowledge of its features. The main contribution of this research is that uses a Software Defined Radio (SDR) system to implement a MCRN in order to measure the behavior of Primary and Secondary signals in both time and frequency using GNURadio and OpenBTS as software tools to allow a phone call service between two Secondary Users (SU). This allows to extract experimental results that are compared with simulations and theory using Rényi-entropy to detect signals from SU in GMSK and OFDM systems. It is concluded that the Rényi-Entropy detector has a higher performance than the conventional energy detector in the Additive White Gaussian Noise (AWGN) and Rayleigh channels. The system increases the detection probability (P_D_) to over 96% with a Signal to Noise Ratio (SNR) of 10dB and starting 5 dB below energy sensing levels.

## 1. Introduction

MCRN have been developed to improve spectrum management for PU given that frequency spectrum holes are used in the transmission of SU [1]. The first task of this technology is to identify said holes within a certain frequency range through one of two alternatives. While one considers that the cognitive system has a priori knowledge of the PU signal, the other alternative consists of a blind process where the signal is unknown [2]. Mobile technologies from 1G to 5G involve different modulation schemes according to the service, distance and SNR of each user. In order to identify the PU signal, a technology must be chosen to determine the frequency range in the spectrum sensing process [3]. Once the frequency range is found, a spectrum analyzer is used to determine the power of the signal. However, since modulation schemes can change over time in certain technologies, this makes it difficult for cognitive radio network systems to distinguish a PU signal from noise [2]. Spectrum sensing mechanisms are used to detect the presence of a PU within the network. This process must be performed in real time so that there is no interference with the services of PU. In GSM systems, Time Division Multiple Access (TDMA) and Frequency Division Multiple Access (FDMA) are used along with a GMSK modulation scheme to allow phone calls and SMS services [3]. In 5G systems, Orthogonal Frequency Division Multiple Access (OFDMA) is a modulation scheme [4,5] based on OFDM. Although these strategies can contribute to the detection of a PU with a priori knowledge of its signal, this signal is a random variable over time and can be considered as a stochastic process. Thus, systems based on a priori information will increase the probability of false alarm (P_FA_) during detection [2].

Many techniques are used in spectrum sensing such as energy detection, cyclostationary detection, matched filters [2,6,7,8,9,10] among others. Matched filters or cyclostationary detection methods need a priori information of the PU signal while others are noise-sensitive as in the case of energy detection [11]. Matched filters and coherent detection are often used to detect a signal by comparing it to a previously known signal (called a template) with the input signal which implies having a priori knowledge of the signal. Matched filter detection is suitable when the transmission of a licensed user includes a pilot, preambles, a synchronization word or spreading codes, which can be used to build a template for spectrum sensing [12]. The energy detection method is susceptible to the uncertainty of noise power and it can only detect the presence of the signal but it cannot differentiate its type thus making it prone to attacks [11,12]. In cyclostationary detection, the signal transmitted by a licensed user is marked by a periodic pattern. This pattern is referred to as cyclostationary and is used to detect the presence of a licensed user. However, the detection process is complex and requires long observation periods to deliver a result [12].

In contrast, cooperative detection is theoretically more accurate because the uncertainty for a single user’s detection process can be minimized through collaboration. However, cooperative approaches cause adverse effects on resource-constrained networks due to the overhead traffic involved in the cooperation and require the use of a Common Control Channel (CCC). This requires creating a frequency channel for the transmission of the CCC [13]. Another approach involves the study of game theory and the impact of resource pricing on the spectrum’s fragility. A NOMA wireless network is implemented, supporting both licensed and unlicensed band access, and used in 5G systems by adopting the principles of Prospect Theory. This research works with licensed and unlicensed users and can be analyzed in MCRN [14].

Some authors analyze signals in the frequency domain and use metrics such as entropy in order to detect PU, rendering detectors less sensitive to noise and increasing the probability of detection (P_D_) [2,7,15,16]. The Shannon entropy metric has been used to detect PU signals in the frequency domain showing better results in terms of noise uncertainly [2,7,15,16]. In contrast, our research analyzes Rényi-Entropy and implements it on a Software Defined Radio (SDR) system through a MCRN protocol that emulates a real system, thus allowing to establish a comparison between simulated and real scenarios. In most of the previous work, results are analyzed in simulation settings, while this is the first research to focus on implementing it on real devices. Rényi Entropy is a measure of uncertainty and a generalized form of Shannon Entropy. Although used in information theory [17,18,19,20], the measured data goes beyond said niche since Rényi-entropy can be applied in economics, finance, machine learning and statistics, among others [17,21,22,23,24]. For instance, Rényi Entropy has been used to distinguish different modulations [25], which is closer to a real MCRN. Compared with Shannon entropy, Rényi entropy can better reflect the difference between two distributions [26].

In this article, we propose to measure the power signal of a PU over time. The Discrete Fourier Transform (DFT) is calculated by translating the signal into the frequency domain while the Rényi Entropy detector is used to find a threshold to differentiate between noise and PU signals. The P_FA_ and P_D_ are calculated for some values of SNR and the results are compared with the work found in literature. We implement the detector on a SDR with a fully operational GSM system that allows a phone call service between two SU and an OFDM transmission system to deliver experimental results.

The proposed component of this research regarding cognition consists in the implementation of a spectrum analyzer to measure the power of the frequency spectrum in real time. The Mobile Cognitive Base Station (MCBS) is implemented on an SDR NI-USRP2922, whose function is to read the power measured by the spectrum analyzer and, if the chosen frequency is available, it is assigned to the SU. If a PU needs to use this frequency, then the MCBS must change the SU frequency to another one within the corresponding range. If no other frequencies are available, the last option is to end transmission for the SU. The experiments include both GMSK and OFDM systems.

The main contribution of this work is to implement a cognitive radio network protocol to enable frequency hopping on an SDR. GMSK and OFDM modulations are used to probe signals from 2G and 5G systems by comparing theoretical results, simulations and implementations. Rényi entropy detect signals from PU to improve the spectrum sensing system. In contrast with the literature, our research implements algorithms and takes real values from the spectrum in MCRN which makes it close to the real environment.

The present paper is organized as follows: Section 2 presents the proposed method used for all experiments. In Section 3, the SDR testbed for experiments is shown followed by the results in Section 4. In Section 5, the discussion is shown and the conclusions are presented in Section 6.

## 2. Rényi Entropy Based Spectrum Sensing

In our region, GSM-850 is used since 2G and 5G technologies coexist in this frequency. A central frequency is used according to the absolute radio frequency channel number (ARFCN) assigned to the operator [3]. In this case, the ARFCN frequency lies in the GSM-850 MHz band [27]. The GSM-850 MHz frequency range was measured and it was found that ARFCN 166, which is 876.8 MHz in Downlink (DL) and 831.8 MHz in Uplink (UL), was free. They were used for experiments, since they are not used by the operator in the laboratory zone. However, if a PU uses this frequency, the cognitive system releases it and changes the frequency of the SU.

A PU signal is considered and described below:

(1) $$y(t)=\{\begin{array}{ccc} n(t) & & noise\\ h(t)*s(t)+n(t) & & PU \end{array}$$

where *n(t)* is noise, *h(t)* is the impulse response of the system, *s(t)* is the signal received from a PU and *y(t)* is the incoming signal comprised of noise and the impulse response [28]. It is considered a system without a priori knowledge of the signal. A binary hypothesis test is used to find out the presence of the PU signal, *H*_0_ is the noise in the absence of a PU signal and *H_1_* indicates the presence of a PU signal [29], as described below:

(2) $$y(n)=\{\begin{array}{ccc} u(n) & & H_{0}\\ s(n)+u(n) & & H_{1} \end{array}$$

where *s(n)* is the PU signal, *u(n)* is the noise and *n* = 0, 1, 2, …, N−1, N is the sample size. It is assumed that the channel has AWGN and the noise has mean of 0 and a variance of 1. The entropy of *y(n*) in the time domain is sensitive to noise uncertainty while the entropy detector in the frequency domain is non-sensitive to noise. Our system is considered to improve the detection of PU presence in the network and operates with low SNR [11]. A Discrete Fourier Transform (DFT) is applied to (2) and (3) is obtained [11]:

(3) $$\bar{Y}(k)=\{\begin{array}{ccc} \bar{U}(k) & & H_{0}\\ \bar{S}(k)+\bar{U}(k) & & H_{1} \end{array}$$

where $\bar{Y}(k)$, $\bar{S}(k)$ and $\bar{U}(k)$ are the complex spectrum of the received signal, PU signal and noise, the DFT size *K = N .H_0_* indicates the absence of a PU signal and *H_1_* indicates the presence of a PU Signal [15].

The input of the system is a random variable since the symbols, amplitude and modulation scheme change over time and users of the cellphone system have a dynamic location and variable speed [3]. If we analyze the signal over time, classic energy detectors are obtained, yet the proposed solutions based on [11] translate the signal into frequency domain and calculate Rényi entropy instead of the Shannon entropy analyzed on [11]. A statistic test also contributes to detect the presence of the PU. Additionally, the designed algorithms were implemented on an SDR as explained in Section 3 to compare results with simulations and theory.

Rényi entropy is described below [30]:

(4) $$H_{\alpha }(Y)=\frac{1}{1-\alpha }\times log\left(\sum_{i=1}^{n}p_{i}{}^{\alpha }\right)$$

Given Y discrete events, *P_i_* is the probability of occurrences in the ith bin, *α* is the Rényi entropy order where *α* ≥ 0 and *α ≠* 1. Using the histogram method to estimate the received signal probabilities, for a given number of bins L, Rényi entropy is estimated by (5) for a statistic test [11]:

(5) $$T(Y)=\frac{1}{1-\alpha }\times log\left(\sum_{i=1}^{L}{\frac{k_{i}}{N}}^{\alpha }\right)\left\{\begin{array}{c} \leq \lambda :H_{1}\\ >\lambda :H_{0} \end{array}\right\}$$

where *N* is the number of samples, the threshold for a Gaussian distribution is described below:

(6) $$\lambda =H_{L}+Q^{-1}(1-P_{FA})\times \sigma_{e}$$

where the Q-function or Complementary Unit Gaussian Distribution Function is described below:

(7) $$Q(x)=\frac{1}{\sqrt{2\times \pi }}\times \int_{x}^{\infty }\exp\left(\frac{-u^{2}}{2}\right)du$$

*H_L_* is the mean value as described below and standard deviation *σ_e_* [11]:

(8) $$H_{L}=\ln\left(\frac{L}{\sqrt{2}}\right)+\frac{\gamma }{2}+1$$

where *L* is number of bins and *γ* is the Euler-Mascheroni constant. In this paper, we choose *α* = 2.5 and 15 bins for test statistics [25].

## 3. Software Defined Radio Testbed for Experiments

**Figure 1:**
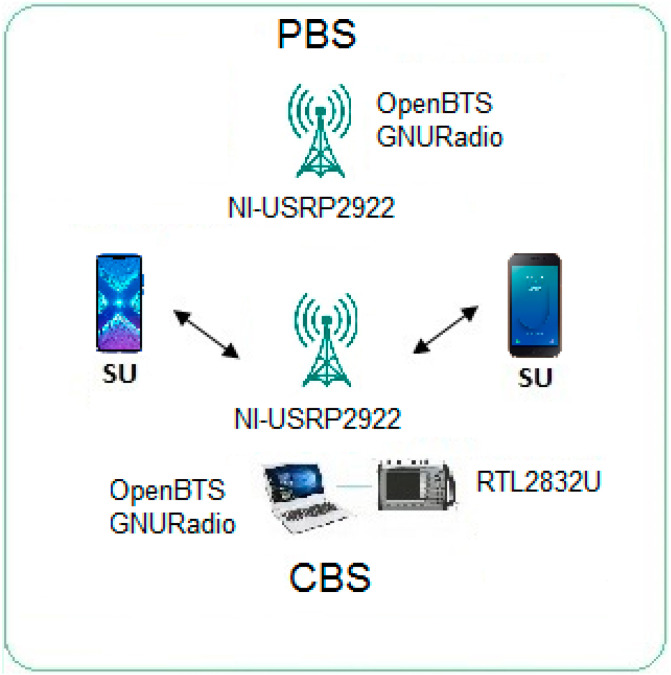
Software Defined Radio Test-bed.

**Figure 2:**
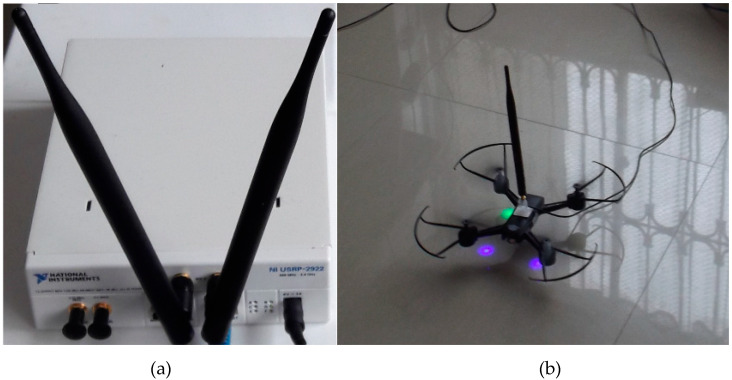
(**a**) Static location Scenario; (**b**) Dynamic location Scenario.

The testbed used for experiments is based on two SDR (NI-USRP2922), one is used for Primary Base Station (PBS) generation and the other one is used for a Mobile Cognitive Base Station (MCBS). A spectrum analyzer RTL2832U is used for spectrum sensing, software systems OpenBTS and GNURadio [31,32,33,34] are installed in the computer that operates the MCBS and a code variation is made to enable a cognitive protocol, so that it can perform permanent spectrum detection and channel assignment to communicate to the SUs. The PBS and MCBS allow the connection with primary and secondary users and the available services are phone calls and short messages services (SMS). Power measurements are taken from the frequency channel when the PUs in motion are connected to the PBS. The testbed is shown in Figure 1. The system considers two PUs and two SUs in communication between them with GMSK or OFDM. During the experiments, the location of the PU can be fixed or dynamic as shown in Figure 2. as well as the location of the SU. User movement is random and changes constantly. The objective is to observe the behavior of Rényi-entropy based on the DFT of the power measurements of any PU present and decide whether a PU signal is present.

The GSM frequency is ARFCN 166 which allows communication in DL and UL. In this case, we use the UL frequency to identify the power of the PU at 831.8 MHz. The RTL2832U is used to constantly measure the frequency spectrum and transmit it to make decision regarding the spectrum.

The experiments are carried out in an indoor environment with elements such as walls, desks, chairs, among others. The distance between the components of the cognitive base station and the PU is 10 m. To carry out the experiments with a fixed PUE, the direct connection of the antenna is established. In the case of a dynamic PUE, the antenna is adapted into a drone that allows its mobility. Only the antenna is moved and not the entire equipment. Figure 2 shows the fixed and dynamic hardware used.

## 4. Results

In this section, the results of the testing scenarios obtained in simulation and the experiments are presented and analyzed.

### 4.1. Simulation Results

In the simulations, Monte Carlo experiments were conducted with the configuration shown in Table 1. The parameters were selected to compare them with the results of an entropy detector found in [11]. A random bits generator was used to generate the date and it is modulated in GMSK. The additional noise is added depending on channel fading (AWGN or Rayleigh). The DFT is then calculated and the histogram method is used to calculate Rényi-entropy. The threshold is calculated depending on the SNR values and figures of the PD, PFA and ROC curves are finally generated. The P_FA_ is an input of the system, the target that P_FA_ will be less than 0.1 (10%) so the P_D_ will be over 90% [11].

The proposed method is compared with energy detection in two possible scenarios: AWGN channel and Rayleigh Channel. The performance of the algorithms is evaluated by calculating receiver operation characteristics (ROC) curves, the P_FA_ and P_D_ over some SNR values.

**Figure 3:**
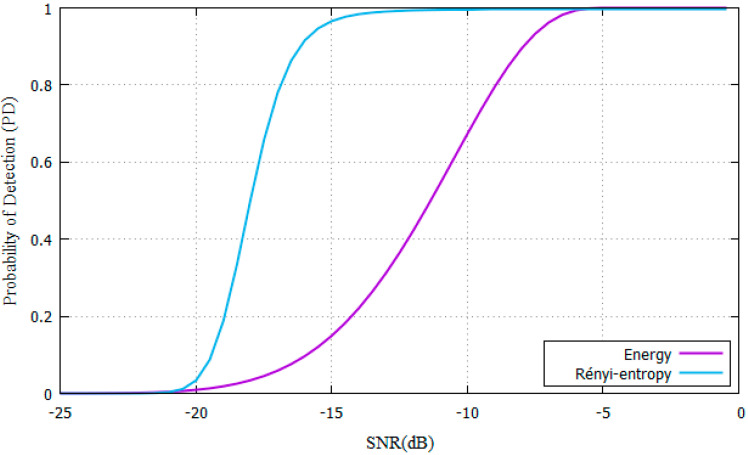
PD vs SNR for Rényi-entropy and energy detector for AWGN channel.

In the AWGN scenario, the parameters shown in Table 1. are used for SNR values ranging from −25 dB to 0 dB. ROC curves are shown in Figure 3. comparing the P_D_ of the proposed Rényi-Entropy detector against the energy detector from [11].

**Figure 4:**
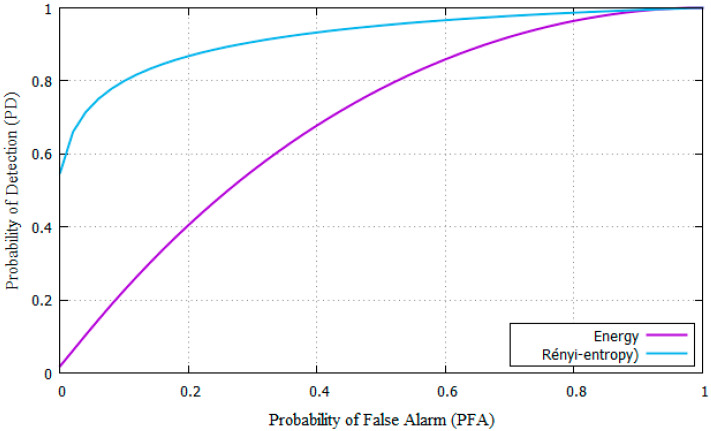
ROC curves for the Rényi-entropy and energy detector for AWGN channel.

Figure 4 shows the ROC curve of P_FA_ versus the P_D_ compared to the energy detection method from [11] for SNR = −18 dB.

The parameters from Table 1. were used for experiments. Results show that the SNR needed is SNR = −16 dB for the proposed Rényi-Entropy detector and SNR= -8 dB for the energy detector. The proposed scheme has an 8dB performance improvement compared with the energy detector in the AWGN channel. As N increases, also does the P_D_ increase, yet it needs more time to make a decision. Samples are stored to compare them with [11].

**Figure 5:**
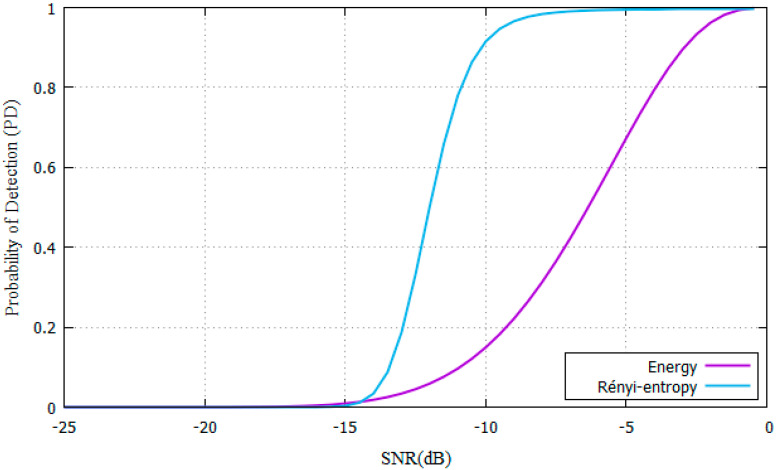
PD vs SNR for Rényi-entropy and energy detector for Rayleigh channel.

The Rayleigh scenario was chosen because it offers the worst performance in detectors [35]. Experiments include SNR values from −25 dB to 0 dB, ROC curves are shown in Figure 5, comparing the P_D_ of the proposed Rényi-Entropy based detector against the energy detector from [11].

**Figure 6:**
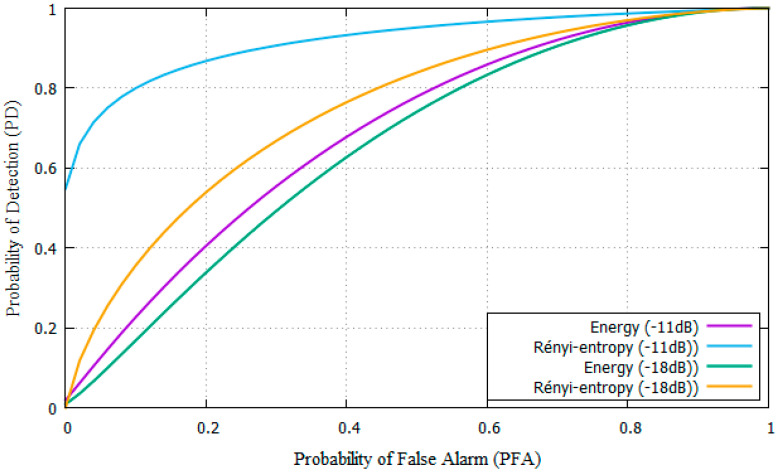
ROC curves for Rényi-entropy and energy detector for AWGN channel with −11 and −18 dB

Figure 6 shows the ROC curve of P_FA_ versus the P_D_ compared to energy detection from [11] for SNR = −18 dB and −11 dB.

To obtain the ROC curves, an SNR must be defined. This value is close to −11 dB in practice. The P_FA_ is an input to the system, the target that P_FA_ will be less than 10% so the P_D_ will be over 90%. The simulation results shows a PFA and PD close to energy detection, but better for Rényi-entropy [11] 

The parameters from Table 1 were used for experiments. Results show that the SNR needed is SNR = −11 dB for the proposed Rényi-Entropy based detector and SNR= −3dB for the energy detector. The proposed scheme has an 8 dB performance improvement compared with energy detector [11].

After comparing the AWGN and Rayleigh channels, a 5 dB difference is noticed because of additional fading losses. The Rényi-entropy detector must have a SNR of −11 dB compared to −16 dB in the AWGN channel. The energy detector must have a high SNR (−3 dB) to detect PU signal with same parameters.

Computational complexity is measured according to [36] by counting the number of real multiplications (RM), real additions (RA) and comparisons. The number of averaged values is noted as *N_AV_*, number of FFT points is noted as *N_FFT_*, the number of samples is noted as *N_S_*, the number of sub-channel is noted as *N_C_* and the number of bins is noted as *L_B_*_._ The number of real operations *C* is described below:

(9) $$C=7N_{AV}N_{FFT}+5N_{FFT}\log_{2}N_{FFT}-\frac{N_{FFT}}{N_{C}}+7L_{B}N_{FFT}-1$$

According to this equation, the complexity indicator is medium at the same level that superimposed training (ST) [37] and energy detector in Frequency [36]. The simple energy detector [11] is classified as low and cyclostationary is high [36]. For the parameters in Table 1, C = 1 × 10^−5^ real operations for the simple detector, C = 3.85 × 10^−5^ real operations for the SCAR-detector [37] and C = 43.5 × 10^−5^ real operations for the Rényi-based detector.

### 4.2. Software Defined Radio Experimental Results

The experiments are implemented on SDR using GMSK and OFDM as follow.

#### 4.2.1. GMSK Experiments

**Figure 7:**
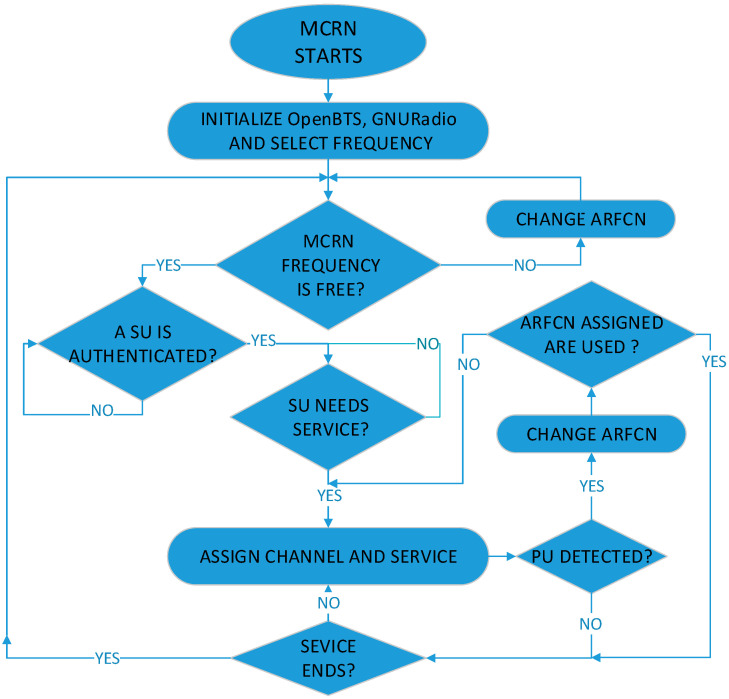
Mobile Cognitive Radio Network Flowchart.

For experimental purposes, a GSM PBS is configured and two PUs are connected via a phone call when PUs are active. The MCBS is configured with a basic cognitive protocol. When the signal is not detected, it will allow two SU to connect through a phone call, but if the system detects a signal with the Rényi-entropy detector, the MCBS must change the carrier frequency. If there is no other carrier frequency available, it must end the call. The flowchart of the system is shown in Figure 7.

The parameters of SDR experiments are shown in Table 2.

**Figure 8:**
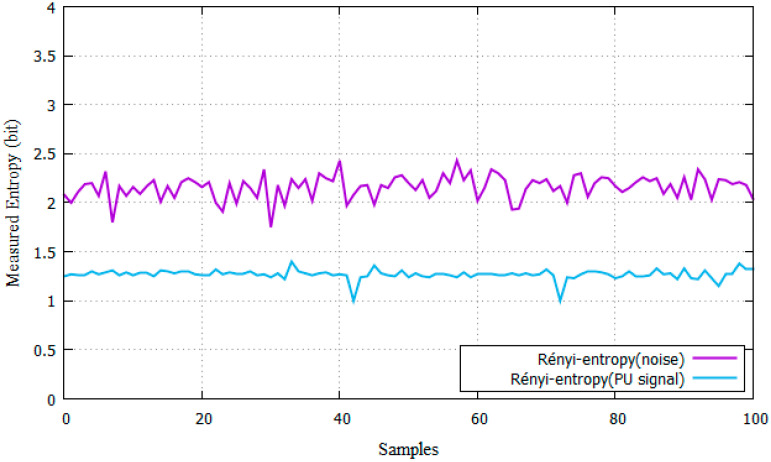
Rényi-entropy of measured PU signal.

At first, there is no ongoing phone call in the MCBS and in the PBS. Then, the average noise power is measured and the noise threshold is set at −90 dBm. Then, the power of the PU is measured when a phone call is taking place between two PUs connected to the PBS. As a result, the power signal is measured and the average SNR of the PU can be computed. The position of the PU is changed and the power and SNR are measured. These data are used to calculate the DFT of the signal and the Rényi entropy to decide if a PU signal is present. An example of the Rényi-entropy results for noise and PU presence can be seen in Figure 8.

The theoretical Rényi-entropy of noise in bits surpasses 2.198 [11]. According to Figure 8, the average Rényi-entropy value for noise is 2.161. Thus, the selected threshold is 2.161 implying that signals above this value are classified as noise. The averaged Rényi-entropy for the PU signal was 1.26. According to the tests, if the Rényi-entropy of the received signal is higher than 2.161, the signal is considered as noise. However, in order to be classified as a PU signal, its entropy value must be remain between 1 and 2.161. The number of detections are compared when a PU is present against the total samples and the P_D_ is estimated.

**Figure 9:**
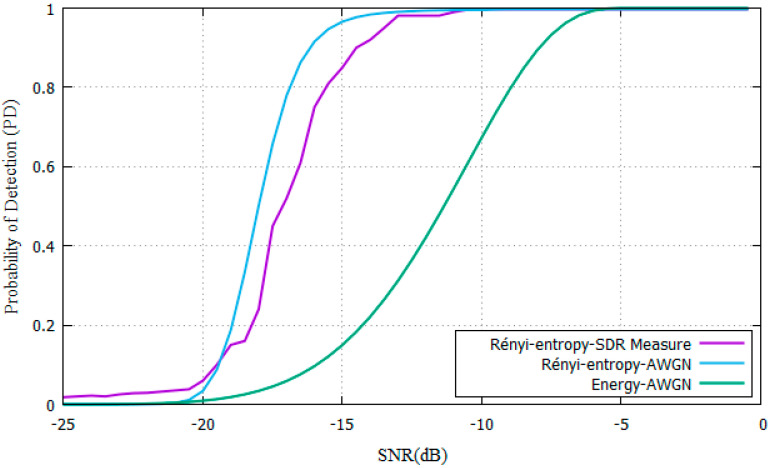
Comparison of GMSK Rényi-entropy on SDR with AWGN simulations.

The results are compared with the simulation results as shown in Figure 9, 600 samples were taken for each SNR value. The experimental results are similar to AWGN model for urban model in an indoor environment with no obstacles.

For the practical measurement of P_D_, two SU remain active and the threshold is assessed. If the results of the Rényi-entropy surpass the SU signal threshold and remain under the noise threshold, then the measured signal is classified as SU. Therefore, the P_D_ corresponds to how many times the SU signal is effectively detected when active and divided by the number of samples. For example, for a SNR = −12 dB in the case of the Rényi-entropy detector, the P_D_ is above 96% (579/600 samples) and the difference with AWGN is equal to −3 dB because due to fading.

**Figure 10:**
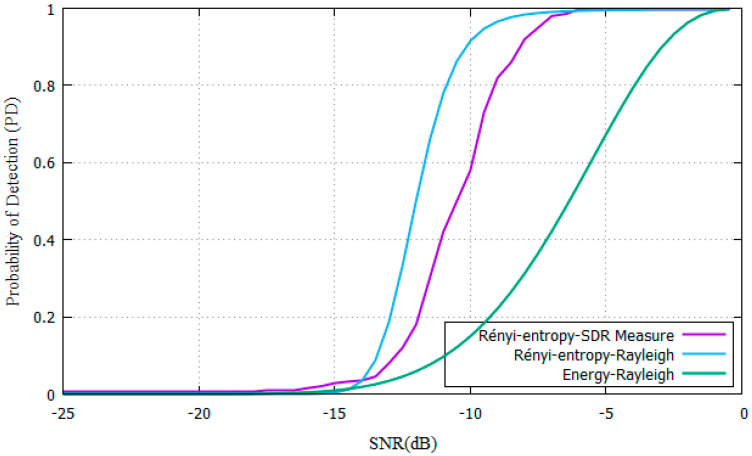
Comparison of GMSK Rényi-entropy on SDR with Rayleigh simulations.

The results are compared with the results from simulations for a Rayleigh channel as is shown in Figure 10. The experimental results prove to be better for an urban model in an indoor environment with obstacles compared to the Rayleigh channel and the difference with AWGN is −3 dB because of fading.

#### 4.2.2. OFDM Experiments

In the experiments, an OFDM transmitter-receiver is configured with a GNU-Radio system and one SU is sending data to another SU. The MCBS is configured with a basic cognitive protocol: when the signal is not detected, it will allow two SU to transmit and receive information, but if the system detects a signal with the Rényi-entropy detector, then the MCBS must change the carrier frequency. If there is no other carrier frequency available, it must cease transmission. The parameters of SDR experiments are shown in Table 3.

The threshold selected for noise is the same as for GMSK with a value of 2.161. Values surpassing this threshold lead to classifying the signal as noise. The averaged Rényi-entropy for the OFDM signal was 1.52. According to tests, if the Rényi-entropy of received signal is higher than 2.161, the signal is labeled as noise, but in order to be classified as a PU signal, its value must remain between 1 and 2.161. The number of detections are compared when a PU is present against the total number of samples and the P_D_ is also estimated.

**Figure 11:**
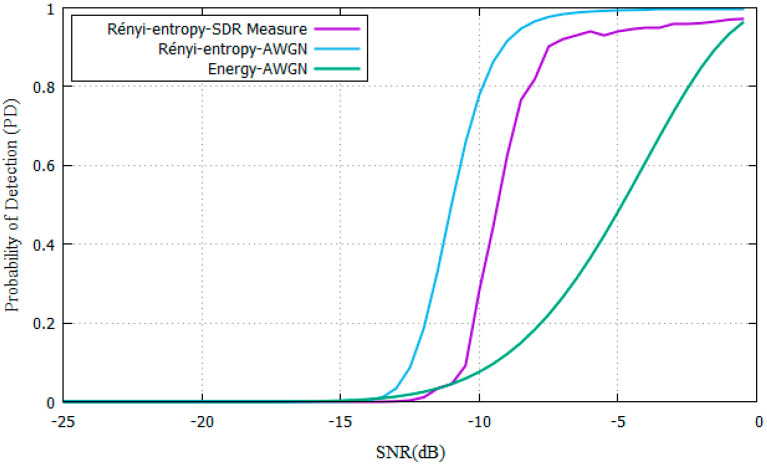
Comparison of OFDM Rényi-entropy on SDR with Rayleigh simulations.

The results are compared with the simulation results as shown in Figure 11. 600 samples were taken for each SNR value. The experimental results are similar to the AWGN model for an urban indoor environment with no obstacles. For example, for a Rényi-entropy detector with SNR = −6 dB, the P_D_ is above 92% (556/600 samples) and the difference with the AWGN model is −3 dB because of fading.

The results show a difference of −6 dB between the GMSK and OFDM experiments. The entropy values change from 1.26 to 1.52, increasing the Rényi-entropy value by 20%. The proposed technique experimental results show a PFA below 10% and PD higher than 90% compared with energy detector in [11].

## 5. Discussion

Simulations were carried out given the same parameters in [2,6,7]. The Rayleigh scenario was included and compared with a practical scenario on a SDR (USRP NI-2922). Results show that the proposed scheme has a better performance compared with the energy detector, by approximately 8 dB for both the AWGN and Rayleigh channels. In experimental results for a selected P_D_ = 90%, this difference is equal to 3 dB in the Rayleigh channel and 5 dB below in the AWGN channel, due to the effects of fading in real scenarios. The experimental average value of noise entropy is similar to the simulation value where the Rényi-entropy of noise stands at 2.198 [11] and the average experimental value of noise is 2.161, showing a difference below 2% in simulations. The averaged Rényi-entropy for the PU signal was 1.26. If the Rényi-entropy value remains between 1 and 2.161 then the signal will be classified as a PU.

## 6. Conclusions

A Rényi entropy-based detector for spectrum sensing was proposed for a MCRN, tested with simulation tools and compared with experiments involving a GSM MCBS and an OFDM implemented on SDR. Simulations were made with Monte Carlo experiments in AWGN and Rayleigh channels. Results show that the proposed solutions outperforms the energy detector method found in literature by about 8 dB. Translating the signal power into the frequency domain decreases the influence of noise and gives better results than other methods, since the detector has no prior knowledge of the modulation or characteristics of the channel.

In SDR experiments, results show that, given a detection target of P_D_ = 90%, the proposed method is superior by 3 dB than the AWGN channel, due to the fading produced in an indoor scenario with obstacles for GMSK. There is a difference of −6 dB with the OFDM system in AWGN fading. The average Rényi-entropy noise is the main variable for threshold selection. In the experiments, it reached 2.161, and, for the PU signal, it was 1.26 for GMSK and 1.52 for OFDM. This significant margin allows the system to detect PU signals and the final results are close to the ones obtained in simulations.

## Figures and Tables

**Table 1:** Simulation Parameters.

Parameter	Description
Number of samples	10,000
PU	2
SU	2
Number of bins L	15
Estimated P_FA_	<0.1
Objective P_D_	>0.9
Signal Noise	AWGN/Rayleigh
Primary Signal	GMSK /OFDM
Bandwidth	12 kHz
Symbol Ratio	10 kbps
Carrier Frequency	40 kHz
Sampling Frequency	100 kHz
DFT size	128
Average Noise Power	−90 dBm
*α*	2.5
Confidence level	95%
Error	5%

**Table 2:** SDR parameters for GMSK experiments.

Parameter	Description
Number of samples	15,000
Number of SU	2
Number of SU	2
Service	Phone Call
Distance of PU to MCBS	0–5 m
Primary Signal	GSMK
Bandwidth	831.8 MHz
Confidence level	95%
Error	5%

**Table 3:** SDR parameters for OFDM experiments.

Parameter	Description
Number of samples	600
Encoded polynomial	[171–133]
Modulation	OFDM
Number of SU	2
Service	Tx-Rx
Distance of PU to MCBS	0–5 m
Number of sub-carriers	64
Bandwidth	831.8 MHz
Confidence level	95%
Error	5%
